# Under‐seen and under‐recognised role of clinical pharmacists in primary dementia care

**DOI:** 10.1002/alz70861_107945

**Published:** 2025-12-23

**Authors:** Nathan Davies, Alice Burnand, Victoria Vickerstaff

**Affiliations:** ^1^ Queen Mary University of London, London, London UK; ^2^ University College London, London, London UK

## Abstract

**Background:**

Most people with dementia are supported at home, primarily by general practice and community‐based primary care teams. Managing dementia in this context presents significant challenges, including complexities of multi‐morbidity and polypharmacy. In response, pharmacists are increasingly providing direct patient care, including roles embedded within general practice, with their scope becoming more clinically focused. Clinical pharmacists, with their specialist expertise in medication management, have emerged as valuable members of the primary care team. However, there is limited research exploring their specific role and experiences in supporting people living with dementia. We aimed to map and understand the clinical pharmacist workforce in UK primary dementia care, focusing on their roles, scope of practice, key responsibilities, and the services they provide. We also explored the experiences of these services from the perspectives of patients, carers, and professionals, identifying opportunities for training and future workforce development.

**Method:**

We conducted a mixed methods study including a national UK survey of clinical pharmacists (*n* =57), semi‐structured interviews with clinical pharmacists (*n* =13), people with dementia (*n* =13), and family caregivers (*n* =15). Survey results were analysed using descriptive statistics, and interviews were analysed using reflexive thematic analysis.

**Result:**

Most pharmacists surveyed (77%) reported working with people with dementia, but many (89%) felt ill‐equipped and expressed a need for more professional training. Interviews revealed that clinical pharmacists are integral members of the primary care team. They offer specialist expertise for this complex patient group—particularly around deprescribing, which was often overlooked and deprioritised by GPs despite its risks. Pharmacists adopted a holistic approach, often working proactively to foresee and minimise risk, addressing the broader needs of people with dementia. However, participants widely agreed that the role of clinical pharmacists remains poorly understood and underutilised in dementia care. Clinical pharmacists often struggled with explaining how their role differed to community pharmacists, and as such were grappling with their professional identify.

**Conclusion:**

Clinical pharmacists play a vital and under‐recognised role in dementia care, providing support that extends beyond medication management to include holistic care and multidisciplinary collaboration. Enhanced training and greater role visibility could help realise their full potential within primary dementia care.

